# Heart failure detection in electrocardiograms using artificial intelligence and pragmatic labelling

**DOI:** 10.1038/s41746-026-02774-4

**Published:** 2026-05-19

**Authors:** Elias Stenhede, Jesper Ravn, Henrik Schirmer, Arian Ranjbar

**Affiliations:** 1 Medical Technology & E-health, Akershus University Hospital, Lørenskog, Norway; 2 Faculty of Medicine, University of Oslo, Oslo, Norway; 3 Department of Cardiology, Akershus University Hospital, Lørenskog, Norway; 4 Institute of Clinical Medicine, Campus Ahus, University of Oslo, Oslo, Norway

**Keywords:** Biomarkers, Cardiology, Computational biology and bioinformatics, Diseases, Health care, Medical research

## Abstract

The diagnosis of heart failure (HF) is resource-intensive, leading to severe underdiagnosis. This study proposes the use of a deep learning model to detect HF solely from electrocardiograms. HF has limited validity in diagnosis codes, lowering the viability of direct application of supervised learning. However, validating the codes with measured levels of circulating N-terminal proB-type natriuretic peptide (NT-proBNP) during training mitigates the impact of label noise. We developed and prospectively validated a neural network for HF detection, using a development cohort of 25,300 patients and an independent test cohort of 43,727 patients. The model achieved an AUC of 0.86 (95% CI 0.847–0.864) for hospital-diagnosed HF, which increased to 0.91 (95% CI 0.900–0.913) with age-adjusted NT-proBNP thresholds and 0.96 (95% CI 0.951–0.963) with stricter limits of 125 and 1000 ng/L. The model was externally validated using the MIMIC-IV cohort of 161,352 patients, yielding AUC values of 0.87 (95% CI 0.861–0.871), 0.90 (95% CI 0.895–0.908), and 0.96 (95% CI 0.950–0.964) with the same labelling strategies. Model predictions were further validated using echocardiographic assessments of diastolic function grade and the H2FPEF score, demonstrating that the model accurately captures both diastolic and systolic function. The model has been released as open source.

## Introduction

Heart failure (HF) is a clinical syndrome characterised by symptoms and/or signs caused by a structural or functional cardiac abnormality and corroborated by elevated natriuretic peptide levels and/or objective evidence of pulmonary or systemic congestion ^1^. Affecting an estimated 1–2% of the global adult population, HF is associated with high mortality rates and substantial societal costs ^2^, and the true prevalence is likely even higher due to significant underdiagnosis ^3,4^.

HF presents with diverse and often nonspecific symptoms, frequently overlapping with those of other conditions, making it challenging to diagnose accurately. It is commonly mistaken for non-cardiac diseases such as respiratory disorders ^5^. Identifying HF typically involves a complex and resource-intensive process, which requires advanced imaging, invasive procedures, and biomarker analysis, making a comprehensive diagnosis inaccessible in regions with limited specialised healthcare. Developing a more affordable and widely available diagnostic method that provides immediate feedback could dramatically improve the care and management of patients with HF.

The electrocardiogram (ECG) is a promising candidate for addressing the diagnostic challenges of HF. Regarded as one of the most versatile and accessible tools, it is utilised extensively across healthcare settings, including the most resource-scarce environments. ECG records the heart’s electrical activity, captured by electrodes attached to the body ^6,7^. Various electrode configurations are available, and the 12-lead ECG is the standard in clinical settings. Apart from being a relatively low-cost tool, its standardised use reduces operator dependency and requires less training compared to methods like echocardiography. However, ECG currently has limited applicability in HF settings, primarily used to identify specific causes of HF, such as atrial fibrillation or old myocardial infarction, with no established diagnostic markers of reduced cardiac function available. On the other hand, if manifestations of HF are present in the ECG, they could potentially be detected by algorithmic pattern recognition.

Computer-aided interpretation of ECGs has a long history, with an acceleration in development during the last decades through the use of machine learning ^8–10^. With the advent of deep learning, several models have been developed to detect conditions such as atrial fibrillation (AF), heart block, valvular heart disease, cardiomyopathy, hypertrophy, left ventricular systolic dysfunction, and myocardial infarction ^11–15^. Although unsupervised methodologies have been attempted, supervised learning is still the predominant methodology. That is, using data paired with ground truth labels to train the models ^9,16^. Due to the lack of a ‘gold standard’ in HF diagnosis, and the inherent observer variability in the current diagnostic methods, the validity of HF diagnosis is known to be limited ^2,17^. There is also a large population of unidentified HF cases, presumably present in most pragmatically collected retrospective data ^2,4^, making supervised learning for HF diagnostics difficult without considerable annotation efforts. To overcome these challenges, previous studies have focused mainly on HF phenotypes with clearly distinct characteristics, particularly heart failure with reduced ejection fraction (HFrEF). Left ventricular ejection fraction (EF) measures the percentage of blood pumped out of the left ventricle with each contraction. Normal values lie in 50–70%, whereas HFrEF typically is defined as EF < 40% ^18^. Previous studies have used varying cutoffs for EF including 35% ^19,20^, 40% ^21–26^ or 50% ^24^. While these results are promising, they exclude a substantial portion of patients with heart failure with preserved ejection fraction (HFpEF), as approximately half of the population has an EF > 50% ^18^.

The demographics of HFpEF differ from HFrEF in that patients are more often female, older, and have more comorbidities ^18^. HFpEF is generally less well understood and particularly underrepresented in AI research. However, recent work has demonstrated that deep learning models trained on ECGs can predict left ventricular diastolic function and elevated filling pressures, an important indicator of diastolic dysfunction and an important indicator of HFpEF, with high accuracy ^15^. Despite this, the reliance on paired ECG–echocardiogram datasets limits the availability of training data for most institutions, particularly those without routine access to large volumes of echocardiographic data.

The objective of this study is to develop a general deep learning–based model that uses ECG data to detect HF across its full range of types, including both preserved, mildly reduced, and reduced EF. The target of interest is HF as a clinical syndrome; however, this condition lacks a definitive ground truth and cannot be directly observed in routinely collected data. Instead, it must be approximated using imperfect proxy labels. We aim to pragmatically collect a large dataset for this purpose, without needing additional annotations. To ameliorate the low validity in ICD-10 HF diagnosis codes, we introduce a labelling strategy including both diagnostic codes and N-terminal pro-B-type natriuretic peptide (NT-proBNP) measurements, a biomarker for HF ^27^.

We hypothesise that, despite substantial label noise, particularly in HFpEF, a model trained on a sufficiently large dataset can learn clinically meaningful patterns and identify patients with HF beyond the limitations of the proxy labels.

## Results

**Fig. 1:**
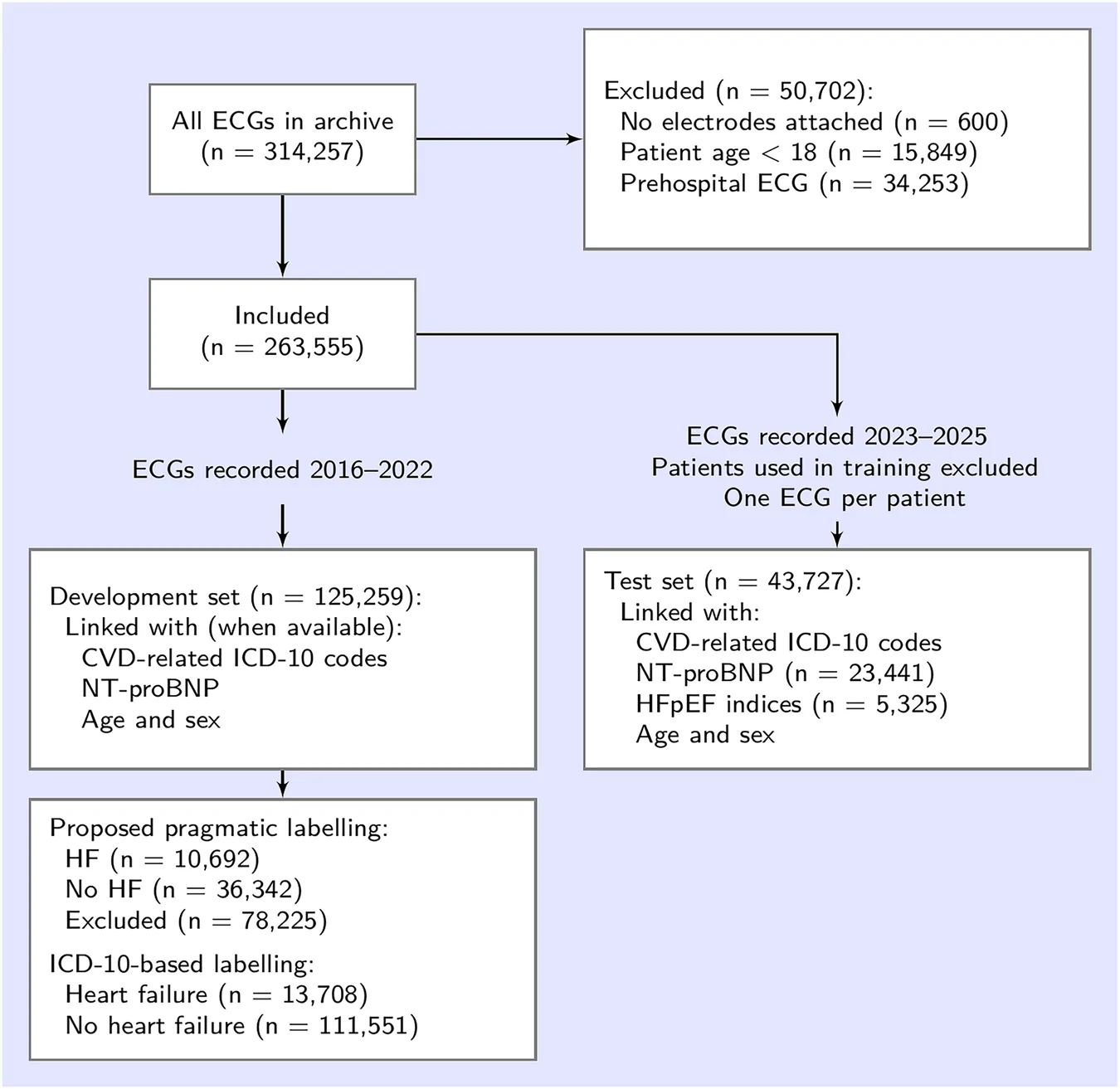
Flowchart of the ECGs used in model development and testing. The labelling of each ECG is determined by whether the patient receives an HF diagnosis within 60 days of ECG recording.

Data for this study were collected from Akershus University Hospital. A development dataset and prospective testing dataset were created by extracting all digital ECGs from the period 2016 to 2025. In total, 314,257 ECG recordings were obtained from 113,963 patients. All ECGs recorded before January 1, 2023, were eligible for inclusion in the model development set. Patients with ECG recordings between January 1, 2023, and May 1, 2025, were included in the test set, excluding those who were part of the model development set to reduce the risk of information leakage. To avoid the disproportionate influence of single patients on the evaluation outcome, only one ECG for each patient was included in the test set. In total, 5325 (12%) of patients in the test set had undergone an echocardiographic examination and had an ECG recorded within 14 days. For these patients, the ECG closest in time to the first echocardiographic examination was chosen to allow for additional analysis in the group, while for the other patients in the test set, the earliest recorded ECG was selected. A flowchart for inclusion is presented in Fig. 1; in total, 125,259 ECGs were included for model development, and after applying the pragmatic labelling strategy, 47,034 ECGs (10,692 labelled HF positive) from 25,300 patients remained.

The age and sex distribution of the development and test sets are presented in Table 1. The test set included patients with a mean age of 65.9 years and 45% women. Known comorbidities of patients in the development and test sets are presented in Table 2. Of the 43,737 patients in the test set, 15,637 (36%) were diagnosed with hypertension, 11,400 (26%) with coronary artery disease, 10,600 (24%) with AF, 6829 (16%) with diabetes mellitus, 5492 (13%) with myocardial infarction, 4766 (11%) with sleep apnoea, and 4280 (10%) with chronic obstructive pulmonary disease. Additionally, 3109 (7%) of the patients received an HF diagnosis within 60 days of their index ECG, and 17,539 patients had their NT-proBNP levels measured in the symmetric 60-day window around their index ECG.

**Table 1:** Age span distribution by gender for patients and ECGs in the development and test sets

	Development set ECGs			Development set patients			Test set ECGs & patients		
Age Group	Female	Male	Total	Female	Male	Total	Female	Male	Total
18–55	6463	6926	13,389	3814	4168	7982	4700	5365	10,065
55–74	8090	13,606	21,696	4498	7151	11,649	6982	10,595	17,577
>75	5458	6491	11,949	2709	2960	5669	8057	8028	16,085
Total	20,011	27,023	47,034	11,021	14,279	25,300	19,739	23,988	43,727

In the test set, only one ECG per patient is included.

**Table 2:** Known comorbidities of patients in the development and test sets

	Development (N=25,300)		Test (N=43,727)		
Condition	N	%	N	%	ICD-10 Codes
Hypertension	8345	33	15,637	36	I1[0-5]
Coronary Artery Disease	8265	33	11,400	26	I251
Atrial Fibrillation	5507	22	10,600	24	I48
Diabetes Mellitus	3838	15	6829	16	E1[0-3], E0[8–9]
Myocardial Infarction	2587	10	5492	13	I21, I22
Sleep Apnoea	2865	11	4766	11	G473
Chronic Obstructive Pulmonary Disease	1837	7	4280	10	J44
Old Infarction	1678	7	2857	7	I252
Chronic Kidney Disease	1554	6	3073	7	N18
Anaemia	951	4	2269	5	D64
Peripheral Vascular Disease	352	1	525	1	I73

For the development set, the table only includes the patients used for model training, and not the ones excluded due to uncertain HF status.

Multiple model architectures were considered, using the pragmatic labelling strategy (3), further described in the Methods section. Among the evaluated architectures, the InceptionTime model consistently achieved the highest validation AUC across all folds. The distribution of AUC values across folds for each model is summarised in Table 3.

**Table 3:** AUC values for each model and fold, the models were trained five times with different initialisation

Fold	Ours	Attia	Cho
1	0.959 ± 0.0010	0.956 ± 0.0028	0.948 ± 0.0070
2	0.964 ± 0.0006	0.962 ± 0.0007	0.945 ± 0.0070
3	0.958 ± 0.0007	0.956 ± 0.0009	0.952 ± 0.0052
4	0.963 ± 0.0007	0.960 ± 0.0006	0.956 ± 0.0036
5	0.962 ± 0.0011	0.958 ± 0.0028	0.955 ± 0.0067

The mean value and standard deviation across the runs are shown.

According to the Wilcoxon signed-rank test, the InceptionTime model significantly outperformed both the Attia ^28^ and Cho ^21^ architectures (*p* < 0.05). For prospective evaluation, five independently trained InceptionTime models were combined into an ensemble, with final predictions obtained by averaging the model-predicted risk (sigmoid-transformed logits). The ensemble outputs a continuous score between 0 and 1, referred to as the model-predicted risk; higher values indicate a greater risk. This final model was then applied to the prospective test cohort for validation.

### Prospective and external validation

Using the test population of 43,727 patients and their corresponding index ECGs, we evaluated performance under three labelling strategies (outlined in Table 4) with strategy (2) being based on age- and sex-adjusted NT-proBNP thresholds defined in Table 5. Because a definitive gold standard for HF was not available, these strategies represent complementary definitions of the same target condition, allowing evaluation across varying levels of diagnostic certainty at the cost of excluding borderline cases. More specifically, labelling strategy (1) reflects routine clinical diagnoses as recorded in ICD-10 codes and therefore captures clinically recognised HF with moderate sensitivity but limited specificity. Labelling strategy (2) incorporates NT-proBNP thresholds to improve diagnostic validity and reduce false positives, approximating HF cases supported by some biomarker evidence. Labelling strategy (3) applies strict NT-proBNP cut-offs to define a high-certainty subset of HF and non-HF patients, but comes at the cost of excluding borderline cases. Accordingly, the model is intended to estimate the relative likelihood of the underlying HF clinical syndrome. The three labelling strategies should therefore be interpreted as alternative operational definitions approximating the same conceptual condition, rather than as separate prediction targets.

**Table 4:** Labelling strategies using defined variables

	Positive label (HF)	Negative label (no HF)
Labelling (1)	HF_curr_	*Φ* HF_curr_
Labelling (2)	HF_ever_ ∧ (proBNP_curr_ > *age*/*sexthreshold*)	*Φ* HF_ever_ ∧ ((proBNP_curr_ < *age*/*sexthreshold*) ∨ *unknown*)
Labelling (3)	HF_curr_ ∧ (proBNP_curr_ > 1000 *ng*/*L*)	*Φ* HF_ever_ ∧ ((proBNP_ever_ < 125 *ng*/*L*) ∨ *unknown*)

HF_curr_ = HF ICD-10 code within upcoming 60 days of ECG; HF_ever_ = any HF ICD-10 code ever; proBNP_curr_ = maximum NT-proBNP within ± 30 days of ECG, proBNP_ever_ = maximum NT-proBNP ever.

**Table 5:** NT-proBNP reference values adjusted by age and sex

Age	Female	Male
18–44	130 ng/L	86 ng/L
45–54	249 ng/L	121 ng/L
55–64	287 ng/L	210 ng/L
65–74	301 ng/L	376 ng/L
≥ 75	738 ng/L	486 ng/L

**Fig. 2:**
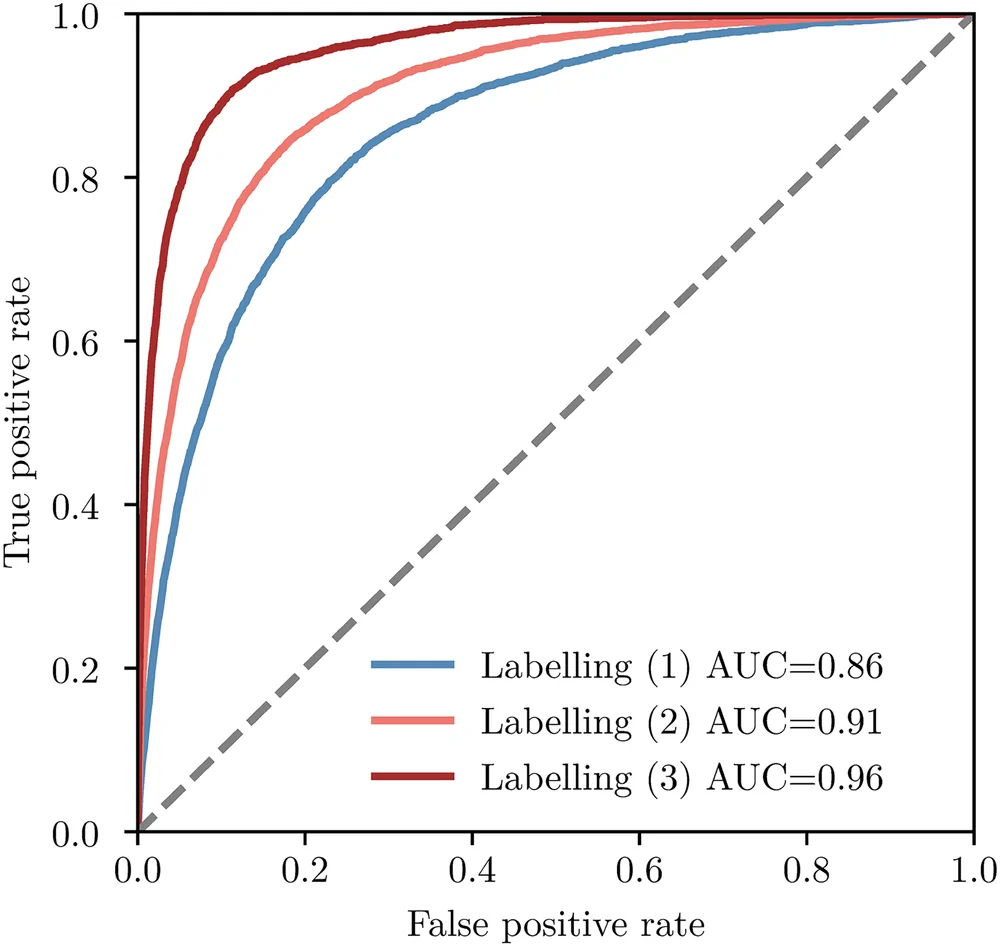
ROC plot for the AI–ECG model evaluated using three different labelling methods. For (1), an ECG is considered positive if the patient receives an HF diagnosis code within 60 days. For (2), the labels are validated by excluding diagnosed patients with levels of NT-proBNP either not measured or not elevated, and non-diagnosed patients with elevated levels, according to age and sex-adjusted thresholds. For (3), labels are validated by only including diagnosed patients with NT-proBNP levels of more than 1000 ng/L and non-diagnosed patients with levels not measured or less than 125 ng/L. The standard errors of the AUC are less than 0.005 for all three labelling strategies.

The AI-enabled ECG model had an area under the curve (AUC) of the receiver operating characteristic of 0.856 (95% CI 0.847–0.864, *N* = 43,727, 3109 positive cases) for labelling strategy (1), 0.906 (95% CI 0.900–0.913, *N* = 34,966; 4098 positive cases) for strategy (2) and 0.957 (95% CI 0.951–0.963, *N* = 21,691; 2,121 positive cases) for strategy (3), as displayed in Fig. 2. Performance against the three labelling strategies stratified by age and sex is shown in Table 6, the model has a higher AUC within younger patient groups. To assess whether including NT-proBNP in labelling for model training was beneficial, an ensemble model trained using ICD-10-based labels only (labelling strategy (1), described in the Methods section) was evaluated on the same three tasks. This model achieved AUC values of 0.855 (95% CI 0.847–0.864, *N* = 43,727, 3109 positive cases) for labelling strategy (1), 0.901 (95% CI 0.894–0.908, *N* = 34,966; 4098 positive cases) for strategy (2), and 0.947 (95% CI 0.940–0.953, *N* = 21,691; 2121 positive cases) for strategy (3). DeLong’s test for correlated AUCs showed no significant difference between models for labelling strategy (1) (*p* = 0.256), whereas strategies (2) and (3) yielded highly significant differences (both *p* < 0.001). It should be noted, however, that these comparisons between labelling strategies do not account for inter-run variability arising from stochastic training effects. All subsequent results relate to the ECG model trained with both ICD-10 codes and NT-proBNP cut-off values.

**Table 6:** The AI-ECG model’s AUC values, along with 95% confidence intervals and sample sizes, are presented when evaluated on subgroups based on age and sex

	Labelling (1)		Labelling (2)		Labelling (3)	
	AUC	N	AUC	N	AUC	N
All < 55	0.88 (0.85–0.91)	10,064 (230)	0.92 (0.90–0.94)	8978 (308)	0.96 (0.94–0.99)	7862 (109)
Male < 55	0.88 (0.84–0.91)	5364 (161)	0.92 (0.90–0.95)	4666 (211)	0.97 (0.94–1.00)	4143 (72)
Female < 55	0.88 (0.83–0.93)	4700 (69)	0.92 (0.88–0.96)	4312 (97)	0.95 (0.90–1.00)	3719 (37)
All 55–74	0.85 (0.84–0.86)	18,828 (1182)	0.91 (0.90–0.92)	15,115 (1489)	0.95 (0.94–0.96)	9477 (686)
Male 55–74	0.85 (0.83–0.87)	11,308 (831)	0.92 (0.90–0.93)	8965 (989)	0.96 (0.95–0.97)	5746 (453)
Female 55–74	0.85 (0.83–0.88)	7520 (351)	0.89 (0.88–0.91)	6150 (500)	0.94 (0.91–0.96)	3731 (233)
All > 75	0.80 (0.79–0.82)	14,835 (1697)	0.86 (0.85–0.87)	10,873 (2301)	0.91 (0.90–0.92)	4352 (1,326)
Male > 75	0.80 (0.78–0.82)	7315 (899)	0.86 (0.84–0.87)	5220 (1254)	0.91 (0.89–0.92)	2175 (675)
Female > 75	0.81 (0.79–0.82)	7520 (798)	0.85 (0.84–0.87)	5653 (1047)	0.90 (0.89–0.92)	2177 (651)

Both the total sample size and the number of HF cases are included.

To further assess generalisability, we externally validated the model on the MIMIC-IV ECG dataset using the same labelling strategies. Taking the index ECG from the 161,352 patients, the model obtains an AUC of 0.866 (95% CI 0.861–0.871) for labelling strategy (1), 0.901 (95% CI 0.895–0.908) for strategy (2), and 0.957 (95% CI 0.950–0.964) for strategy (3).

The specificity, sensitivity, positive and negative predictive values, and total number of predicted positives at various thresholds are presented in Table 7. The model has a high negative predictive value, ranging from 0.99 at a threshold of 5% model-predicted risk to 0.94 at a threshold of 95% model-predicted risk.

**Table 7:** Threshold-wise performance on the Ahus and MIMIC-IV test datasets, evaluated using labelling strategy (1), i.e., if the patients received an ICD-10 code for HF within 60 days or not

	Ahus (N=43,727, cases=3109)					MIMIC-IV (N=161,352, cases=7539)				
Thr.	Sens.	Spec.	PPV	NPV	Pred+	Sens.	Spec.	PPV	NPV	Pred+
0.05	0.90	0.62	0.15	0.99	18,134	0.92	0.59	0.10	0.99	69,928
0.25	0.75	0.80	0.23	0.98	10,286	0.77	0.81	0.17	0.99	34,680
0.50	0.60	0.89	0.29	0.97	6463	0.63	0.90	0.23	0.98	20,821
0.75	0.43	0.94	0.37	0.96	3677	0.45	0.95	0.29	0.97	11,873
0.95	0.17	0.99	0.50	0.94	1023	0.19	0.98	0.37	0.96	4002

The relatively low positive predictive value is consistent with heart failure being underdiagnosed.

*Sens*. Sensitivity, *Spec*. Specificity, *Thr*. Threshold, *PPV* Positive Predictive Value, *NPV* Negative Predictive Value.

**Fig. 3:**
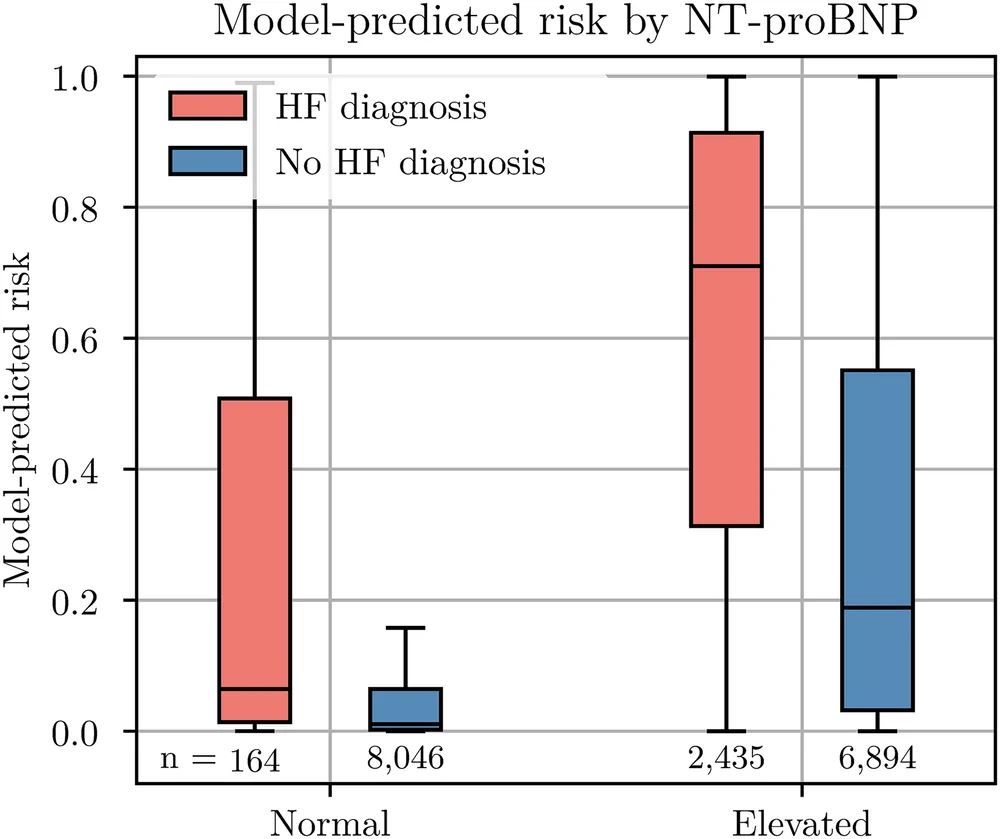
Patients with elevated NT-proBNP levels show higher model-predicted risk of HF (*p* < 0.001), especially among those with a diagnosis of HF in the form of a registered ICD-10 code. The model also identifies increased risk in patients with elevated NT-proBNP who lack a formal HF diagnosis.

By applying the current guidelines for age and sex adjusted cut-off values for NT-proBNP, shown in Table 5, we group all patients by diagnosed HF or not, as well as by elevated NT-proBNP or not in Fig. 3. The model discriminates well between patients with normal levels and no diagnosis and those with abnormal levels and a diagnosis, achieving an AUC of 0.823 (95% CI, 0.813–0.834).

**Fig. 4:**
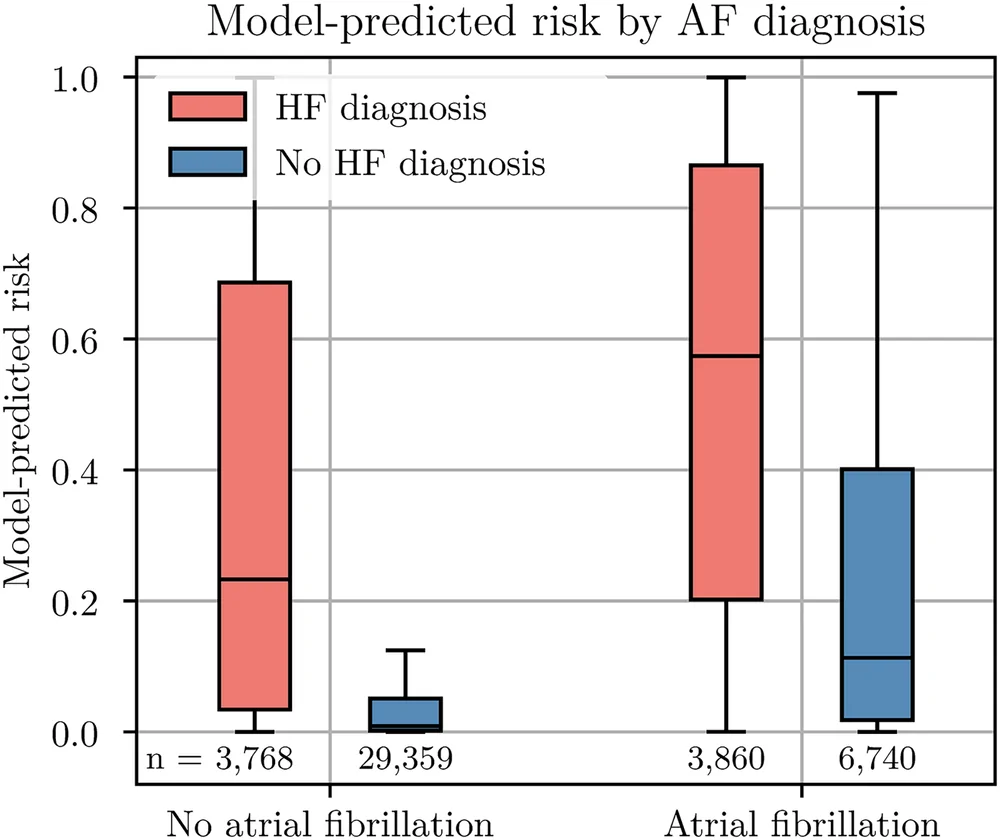
Patients diagnosed with atrial fibrillation have a higher model-predicted risk of HF than those without AF (*p* < 0.001). Patients formally diagnosed with AF but not HF have a higher model-predicted risk than those diagnosed with neither, suggesting the model captures comorbidity-related risk.

As AF is known to cause elevated levels of NT-proBNP, we further stratify the full group by diagnosed AF or not in Fig. 4. The AF group is considerably older, with a mean age of 75.1 years, compared to 62.9 years in the non-AF group. When evaluated against labelling strategy (1), the AUC of the model was 0.857 (95% CI 0.845–0.869), and in the AF group, the corresponding AUC was 0.778 (95% CI 0.764–0.792).

**Fig. 5:**
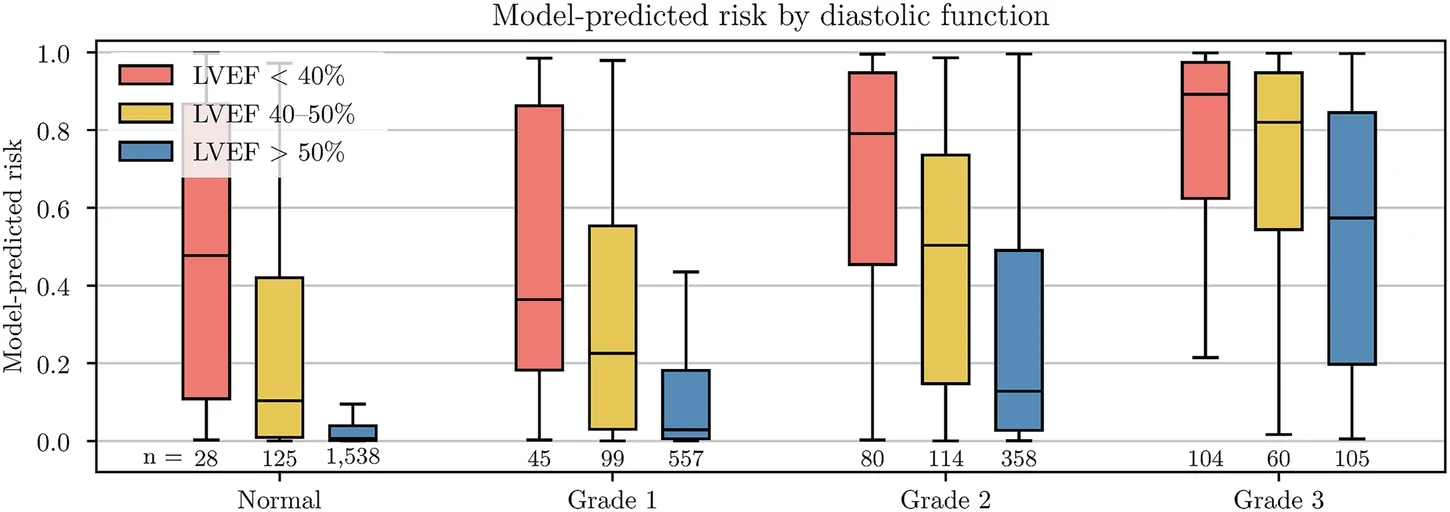
Model-predicted HF risk increases with worsening diastolic function. Within each diastolic function grade, the model assigns a higher risk to patients with lower EF. Among patients with preserved EF, the model still identifies elevated risk with an AUC of 0.800 (95% CI 0.782–0.819) for detecting grade 2/3 diastolic dysfunction, supporting its ability to detect HFpEF-related phenotypes. The model consistently assigns low risk to patients with good diastolic function and EF > 50%, discriminating against the other groups with an AUC of 0.828 (95% CI 0.814–0.842).

Using the subpopulation of 5340 patients who underwent an echocardiographic examination and recorded an ECG within 14 days, the results could be further analysed using the diastolic function grade as calculated from echocardiography ^29^. We stratified the subgroup based on both left ventricular EF and diastolic function grade. In total, 4463 patients had sufficient parameters to calculate diastolic function. Of these, 1,691 (39%) had normal function, 701 (16%) had grade 1 dysfunction, 552 (13%) had grade 2 dysfunction, 269 (6%) had grade 3 dysfunction, while 1245 (26%) had indeterminate diastolic function and were thus excluded. The resulting stratified model-predicted risk is shown in Fig. 5. The model consistently assigns low risk to patients with normal diastolic function and high EF, achieving an AUC of 0.828 (95% CI 0.814–0.842) for discriminating between normal diastolic function and EF > 50% against the rest. Using the optimal threshold determined by Youden’s index (0.63), the sensitivity was 0.71, and the specificity was 0.80. The model assigns a higher risk to patients with lower EF, but still high risk to patients with preserved EF and worse diastolic function, suggesting the model-predicted risk is an indicator of severity. Within the subgroup with EF > 50%, the model distinguished normal/grade 1 from grade 2/3 diastolic dysfunction with an AUC of 0.800 (95% CI 0.782–0.819). Using the optimal threshold determined by Youden’s index (0.57), the sensitivity was 0.82 and the specificity was 0.66.

**Fig. 6:**
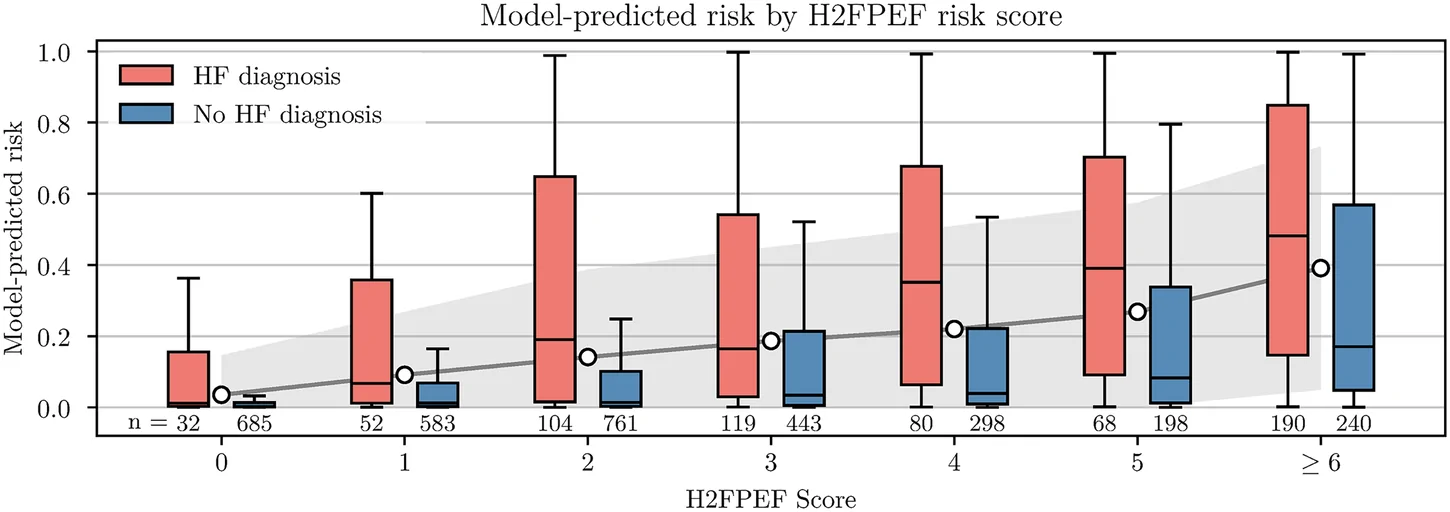
Model-predicted risk for patients who have undergone an echocardiographic examination and estimated EF > 50%, stratified by H2FPEF score and HF diagnostic code. In every risk category, the model assigns a higher median risk to patients with an HF diagnosis (*p* < 0.001). The model-predicted risk also increases monotonically with increasing H2FPEF score. In the H2FPEF risk model, a score of 6 or more corresponds to a greater than 90% risk of HFpEF. The line with circular markers corresponds to the mean model-predicted risk across the H2FPEF groups, and the shaded area represents the standard deviation, regardless of diagnosis.

Model-predicted risk for the entire subset with EF > 50% was further stratified based on the H2FPEF score (Table 8), as shown in Fig. 6. In total, 3860 patients had EF > 50% as the necessary estimated parameters. The results reveal that individuals with higher H2FPEF scores also had higher model-predicted risk. Within each H2FPEF stratum, patients with an HF diagnosis had significantly higher predicted risk compared with those without HF (Wilcoxon rank-sum test, all *p* < 0.001), suggesting that the model provides additional discriminative information for assessing HFpEF risk.

**Table 8:** The H2FPEF scoring system used in this study

Clinical variable	Criteria	Points
**H**eavy	Body Mass Index > 30 kg/m^2^	2
**H**ypertensive	Any I{10–15}.* excluding I11.0	1
Atrial **F**ibrillation	Any I48.*	3
**P**ulmonary hypertension	Pulmonary artery systolic pressure > 35mmHg	1
**E**lder	Age > 60 years	1
**F**illing Pressure	Doppler echocardiographic E/e’ > 9	1
**H2FPEF score**		**Sum (0–9)**

Hypertension is identified via ICD-10 instead of 2 or more antihypertensive medicines.

## Discussion

This study presents a labelling strategy and a corresponding AI–ECG model capable of general HF detection. The model is evaluated prospectively in two main ways.

First, the model is evaluated on the full test set to predict all HF irrespective of EF and diastolic function grade. We show that AUC increases significantly when NT-proBNP is used to validate the ICD-10 codes. When both the proposed model and the model trained using ICD-10-based labels are evaluated against ICD-10–based labels, the difference between training strategies is modest, which could reflect the limited validity of ICD-10 codes as a proxy for the HF syndrome, resulting in an upper bound on performance under this evaluation definition. In addition, the model trained using ICD-10–based labels was exposed to a substantially larger number of ECG recordings, which may partially offset the impact of increased label noise. The benefit of incorporating NT-proBNP into the labelling becomes more apparent when the evaluation labels are also refined using NT-proBNP thresholds, where the stricter definition better approximates the underlying clinical condition, by excluding borderline cases. The AUC values are 0.957 vs 0.947, which could be clinically meaningful. Stratification by age group and sex shows that the AUC for the proposed model is inversely correlated with age. This effect might come from elevated label noise, which tends to increase with age, driven by a higher prevalence of HF and comorbidities ^4^. Despite this, the model shows strong performance across all demographic groups, suggesting that it captures HF-specific signs in the ECG rather than age-related signs only. Additionally, the population is split into patients with an AF diagnosis or not, showing that the model assigns a higher risk in the former group, which reflects the fact that AF and high age are risk factors. We also show that the model further differentiates risk after stratification by NT-proBNP levels according to current guidelines. With the same labelling strategies, we also evaluate the model on the MIMIC-IV dataset collected at Beth Israel Deaconess Medical Center in Boston, MA. The results are in line with our prospective validation, with a slightly higher AUC for labelling strategy (1) and no significant difference between labelling strategy (2) or (3). As the labelling strategy (1) relies on ICD-10 codes alone, this difference might stem from differing ICD-10 reliability between Beth Israel Deaconess Medical Center and Akershus University Hospital. Second, we evaluate the model using the diastolic function score and EF. The model assigns very low risk to patients with normal diastolic function and EF > 50%, as this is the group with the least signs of HF. As HFpEF is considered the most challenging phenotype, the subgroup is further categorised and analysed using the H2FPEF risk score, and split by ICD-10 HF diagnosis or not. In all H2FPEF strata, the model assigns a higher risk to patients with an ICD-10 HF diagnosis. For patients with low H2FPEF scores and no HF diagnosis, the model consistently assigns low risk, as is expected. The model assigns relatively higher risk to patients with HFpEF score ≥3, suggesting either that the model captures comorbidity-related risk or that there are significant numbers of undiagnosed patients with H2FPEF in these groups.

To verify whether there are significant numbers of undiagnosed patients in the test set, we perform a small-scale qualitative assessment of patients. The qualitative assessment was performed by sampling patients based on model-predicted risk and additional clinical data. In total, 60 patients with an EF of greater than 50%, measured NT-proBNP levels of less than 125 ng/L, and no ICD-10 HF diagnosis code. Group (a) comprised the 30 patients with the highest model-predicted risk, and Group (b) the 30 with the lowest model-predicted risk. In addition to evaluating label quality, these groups were selected to assess the model’s capability of generalising to HFpEF with low NT-proBNP levels, presumably the most challenging cases to label automatically. These scenarios would have been labelled as no HF in all the suggested labelling strategies. The cases were evaluated by a senior cardiologist blinded to both the sampling procedure and model prediction. The cardiologist had access to all available information about the patient, including unstructured data and information recorded by other care providers. In group (a), 24 out of 30 patients had HFpEF or showed strong signs of HFpEF (either enlarged left atrium, increased filling pressures, or both), while six patients did not show strong signs of HFpEF. Group (b) included 27 patients without HFpEF and 3 patients with HFpEF (in addition to the criteria above, one had left ventricular hypertrophy due to hypertension). A short description of each case is provided in the Supplementary Table 2. These results highlight the challenge with automated data annotations, especially as many patients with complex phenotypes, such as HFpEF, go unnoticed and thus are not possible to label using ICD-10 codes. Furthermore, this illustrates that although the model was trained using highly purified labels, requiring elevated NT-proBNP levels for positive labelling, it is still capable of detecting HF in some cases where NT-proBNP levels are low.

In contrast to previous work, the model proposed in this study aims to detect *hospital-diagnosed* HF rather than specific echocardiographic parameters such as reduced EF or left ventricular diastolic function. Previously proposed models have not been made publicly available due to privacy concerns, making direct comparisons difficult. Nevertheless, we briefly summarise the main results from prior studies. Among models trained to classify EF < 35%, reported AUC values range between 0.89 and 0.93^19,20^. For EF < 40%, Cho et al.^21^ report AUC values between 0.913 and 0.961, Jentzer et al.^22^ report 0.83, Kwon et al.^24^ 0.843 and 0.889 for internal and external validation, Attia et al.^25^ 0.95, and Sangha et al.^26^ 0.91. For EF < 50%, Kwon et al.^24^ report 0.821. In classifying left ventricular diastolic dysfunction, Lee et al.^15^ report AUCs of 0.847, 0.911, and 0.943 for diastolic dysfunction grades ≥1, ≥2, and 3, respectively. Overall, previous results are strong but vary substantially across studies. We believe that this variability primarily reflects differences between validation cohorts, as differences in model architecture or training are important, but the expected difference in AUC should be less than what is observed, given reasonable training choices. For example, our re-implementations of two of the aforementioned models and training recipes resulted in drops in the AUC of up to about 0.01. This highlights the need for evaluation on public ECG datasets, and, ideally, the sharing of full models to enable later comparisons. Our results are consistent with what could be expected given the aforementioned studies. A model trained to predict hospital-diagnosed HF could be expected to perform slightly worse in terms of AUC than models specialised for a single echocardiographic parameter, but better when integrating multiple pathophysiological aspects, as illustrated in Fig. 5. For example, our AUC of 0.828 can be compared to the AUC of 0.911 for detecting diastolic dysfunction grade ≥2 reported by Lee et al.^15^. However, our model additionally separates each diastolic function grade by EF, providing additional clinical utility.

The model-predicted risk could serve as an additional screening tool, similar to NT-proBNP. A major advantage is that test results are non-invasive and instantly available after taking an ECG. As ECGs are already readily utilised in the clinic, screening using the model would not necessarily take extra time or resources, given that the infrastructure for digital ECGs is in place. Further, AI–ECG captures electrophysiological traits of HF, whereas NT-proBNP is secreted in response to myocardial stretch. Consequently, the AI–ECG and NT-proBNP may provide complementary information, and AI–ECG may identify risk patterns not reflected in NT-proBNP levels.

While results at various decision thresholds are presented in Table 7, the model should not be endowed with a fixed, universal threshold. Optimal decision thresholds are context-dependent and should be defined according to the intended clinical use. For example, in a primary care screening setting, a medium-high model-predicted risk could indicate the need for NT-proBNP testing, whereas a high risk might justify both NT-proBNP measurement and referral for echocardiography. Conversely, in hospital settings such as respiratory wards, where NT-proBNP is often measured routinely, combining model predictions with NT-proBNP could help prioritise echocardiographic evaluations or guide early treatment initiation. However, these potential implementations remain hypothetical. During model development, ranking was prioritised over absolute calibration to maximise diagnostic discrimination, and area under the receiver operating characteristic curve was the main metric used during cross-validation because it directly reflects ranking performance, that is, the probability that a positive case is ranked higher than a negative case. Therefore, the model-predicted risk should be interpreted as a ranking metric rather than a calibrated probability of HF. In practical terms, a higher score indicates that the ECG is more consistent with HF-related patterns, but the numerical value itself should not be interpreted as the absolute probability that the patient has HF. Calibration-related behavior is presented in Supplementary Fig. 4, but should be interpreted with caution because ICD-10-coded HF is a noisy proxy for the underlying HF syndrome. The value of a predictive model lies not only in predictive accuracy but in its ability to improve patient outcomes. The real-world downstream consequences of threshold selection on patient outcomes remain unknown. The answers to such questions must be established through prospective clinical studies or, where possible, retrospective outcome analyses.

While one of the main contributions of our work is to enable model training in the presence of uncertain labels, such uncertainties remain a significant challenge when evaluating the model on the test set, as no gold standard labels exist within the hospital. The true HF status remains unknown for many patients in the full dataset, but can be approximated to a better degree in the subgroup who underwent echocardiographic examination. Still, even in this group, the model identified HF patients with normal EF, normal NT-proBNP levels, and no diagnosis. In such cases, HF could potentially be concluded by inspecting the unstructured journal text and echocardiographic recordings, highlighting the need for larger clinically validated datasets for model evaluation. Even when evaluating against clinically validated datasets, HFpEF classification in particular involves inherent ambiguities due to the dependence on subjective interpretation and statistical risk models such as H2FPEF. Another source of label noise stems from data collection, as patient data collected outside the hospital context is sometimes not available. Further, in the diastolic function and H2FPEF grading algorithms, imputation was performed for missing values. Specifically, at most one out of the parameters was allowed to be missing, and in such cases, it was set to zero. This may have resulted in the H2FPEF grade being underestimated by one point for some patients, and for others, it may have led to their movement in or out of the indeterminate diastolic function category. The Ahus cohort used for this study includes a relatively broad sociodemographic spectrum, and while the model has been prospectively validated on data collected in 2023–2025, some of the analyses conducted in this study were specified post hoc. The model was also externally validated on the MIMIC-IV-ECG database, collected in a different sociodemographic setting. Despite this, it should be noted that less than 20% of the background population is of non-Caucasian descent.

Using routinely collected data like blood samples and ICD-10 codes is a promising direction to build holistic risk detection models, perhaps in combination with more specialised models. Future work should thus explore various labelling strategies, such as including more diagnostic codes and blood tests during training. Besides considering more biomarkers, it is plausible that adding echocardiographic information in model training will improve performance and utility. For example, a model trained to both estimate EF and classify HF from ECG might indicate the suspected HF phenotype. It would also be of interest to investigate which pathophysiological traits the model captures through post-hoc analyses of correlations between model risk and an extended set of echocardiographic parameters.

## Methods

This paper presents a predictive modelling study using retrospective data and a prospective model validation. The study design received ethical approval from Helsedirektoratet (case 21/39600–3) on 2022-07-01, with an update to include additional data (case 24/45684–5). In particular, exemption from consent was applied following the Norwegian Health Personnel Act, Chapter 5, Section 29 ^30^, through the use of algorithmic data mining and anonymization ^31^.

The study aimed to develop an ECG classification model for heart failure, based on deep learning. In particular, by pragmatically collecting a large dataset without the need for additional expert annotations. The following sections describe how the development dataset was built, the machine learning methodology, and the prospective validation setup. Both data for development and clinical validation were collected at Akershus University Hospital, a healthcare network comprising four tertiary hospitals (Ahus Nordbyhagen, Ahus Gardemoen, Ahus Kongsvinger, and Ski Hospital), providing service to more than 10% of the Norwegian population. The service area covers both urban and rural areas, providing a population with a broad sociodemographic background.

### Primary and secondary outcomes

The primary outcome of this study was to assess the performance of the AI model to identify patients with HF, here defined as any ICD-10 code under I42.*, I50.*, or I11.0. As part of this analysis, the model’s performance was compared against NT-proBNP, a widely used biomarker for HF detection. The secondary outcome focused on identifying patients with heart failure with preserved ejection fraction (HFpEF), a subgroup of HF patients that remains underdiagnosed and poorly understood.

### Development dataset

Data mining was done via a research platform connected to the internal picture archive and communication system (PACS) and electronic health record (EHR) of the hospitals^31^. To build the development dataset, a retrospective cohort was used, comprising all ECGs recorded between 2016 and 2022. For the corresponding patients, ICD-10 codes for heart disease and, if available, NT-proBNP measurements were collected.

Extracted 12-lead ECGs were recorded with sampling frequency of 1000 Hz or 500 Hz, all with a length of 10 s, from all hospital departments. The multivariate time series data, stored in the DICOM standard, were retrieved from the PACS. Pre-processing was done by removing metadata and reformatting the time series into a consistent representation by downsampling the 1000 Hz recordings to 500 Hz, and z-normalising per-lead. In everyday practice, NT-proBNP tests were analysed in-house by the hospitals, carried out using electrochemiluminescence immunoassay (ECLIA) and the sandwich principle, from Roche Diagnostics (cobas pro e801 at Ahus Nordbyhagen and cobas pure e402 at Ahus Kongsvinger). The test results were retrieved from the EHR and securely transferred to a research platform, along with diagnosis codes for heart conditions, as well as patient age and sex. To ensure anonymity, patient and ECG identifiers were replaced with generated identifiers, and all dates were adjusted by random offsets. This preserved the relative timelines while protecting patient privacy. Sex was defined by the value reported in the DICOM file corresponding to each ECG.

### Inclusion and labelling strategy

The main challenge in developing a supervised deep learning model for HF is defining the ground truth. Diagnostic codes, such as ICD-10, are known to have low validity for HF. To get better accuracy in the labelling, NT-proBNP measurements were used in conjunction with ICD-10 codes to validate the condition.

NT-proBNP is a prohormone and one of the most used biomarkers for HF, measured via blood samples. NT-proBNP is particularly useful for ruling out HF, and recommended decision limits are set low to give a high predictive power of a negative test. In patients without acute symptoms, the threshold 125 ng/L is recommended to rule out HF. For patients with acute dyspnoea or other acute symptoms, the recommended level is 300 ng/L ^18^. As we do not have access to information about symptoms during labelling, NT-proBNP levels of less than 125 ng/L were used to ensure the patients without diagnosis were indeed, with high probability, free of HF. While low levels are used to conclude the absence of HF, elevated levels may occur due to a multitude of cardiac or non-cardiac causes, such as AF, Chronic obstructive pulmonary disease (COPD), and renal dysfunction. Moreover, higher levels are usually observed with female sex, increasing age, and lower body mass index (BMI), which further complicates interpretation. NT-proBNP has also been investigated in the setting of confirming acute HF, with thresholds between 450 and 1800 ng/L suggested being dependent on age only ^32^. Determining a cut-off for affirming HF diagnosis is a trade-off between reducing the number of erroneously diagnosed patients and keeping more patients with HF in the development set. As one of the main goals is to be able to identify patients with HFpEF and HFmrEF, groups where increased levels of NT-proBNP are less pronounced than in HFrEF^33^, an age-independent cut-off for affirming HF diagnosis was set at 1000 ng/L. We hypothesise that this threshold results in a development cohort containing sufficient examples of HFmrEF and HFpEF.

For each ECG, all heart-related ICD-10 codes and NT-proBNP measurements, with corresponding time stamps, from the same patient were collected. Specifically, ICD-10 codes I42.*, I50.* and I11.0 were used to identify HF. The diagnosis codes and NT-proBNP measurements were then aggregated on three levels. For diagnostic codes, we checked for HF or related CVD within a 60-day window after the ECG recording. Additionally, prior or future HF or CVD were denoted outside the time window. Similarly, peak NT-proBNP measurements were recorded within 30 days before and after the recording.

The following algorithm was then used to label and include ECGs in the development dataset. For each ECG:

- The ECG was included and considered to be retrieved from a patient with HF if the subject received an HF diagnosis code within 60 days of the ECG date and had a peak NT-proBNP measurement of more than 1000 ng/L within a 60-day window centred on the ECG date (30 days before to 30 days after).
- The ECG was included and considered to be from a patient without HF if there was no prior or future HF diagnosis code and no NT-proBNP measurement of more than 125 ng/L either historically or up until the date of dataset extraction.
- The ECG was excluded if it did not fulfil any of the two above conditions.

This corresponds to labelling strategy (3) in Table 4. The definition results in a relatively purified HF-negative class, as patients with any prior HF diagnosis or elevated NT-proBNP measurements are excluded. While this asymmetry reduces label noise and improves the reliability of the training labels, it may produce a negative cohort that is cleaner than typical clinical populations, where borderline or uncertain cases are common.

### Model architecture and training

To train and evaluate the models, we used 5-fold cross-validation, where the dataset was divided into folds at the patient level to minimise the risk of data leakage between folds. Three distinct model architectures were considered, two of which were previously used to classify HF. The two previously proposed models, referred to as Cho ^21^ and Attia ^19^, based on the name of the first author, were reimplemented to the best extent, according to the description in the respective publications. In addition to these two models, a model based on the InceptionTime architecture ^34^ was evaluated. InceptionTime-based models have shown promising results in ECG classification ^35^. The models took only ECG time-series as input; no additional clinical or demographic variables (e.g., age or sex) were provided to the network.

The standard 12-lead ECG format contains redundant information, as two of the six limb leads are sufficient to reconstruct the rest using Einthoven’s Law. Therefore, only eight leads were used to develop the InceptionTime-based model (specifically, aVL and aVF, as well as V1–V6). Sample rates of either 100 or 500 Hz, with the higher rate improving performance across all models except InceptionTime. This discrepancy was attributed to InceptionTime’s limited receptive field, as it lacks downsampling layers. To address this, we prepended one to three blocks of [Conv(kernel_size=5, depth=32, stride=2), BatchNorm, GELU] to expand the receptive field. The configuration with two blocks consistently achieved the best results and was used for all subsequent experiments.

Hyperparameters were optimised using the maximum validation AUC achieved during training as the selection criterion. After the optimal configuration was identified, each model was retrained, and the weights corresponding to the highest validation AUC were retained for evaluation.

The final model was an ensemble of five independently trained InceptionTime networks. Each ensemble member was trained on the same development data but with a different validation fold in the five-fold cross-validation scheme and a different random weight initialisation. Predictions were obtained by averaging the model-predicted probabilities from each member. This approach mitigates the risk of one model dominating the ensemble output while leveraging the known generalisation and stability benefits of ensembling ^36^.

The InceptionTime model configuration is shown in Table 9. To update the model weights, the Muon optimiser was used for multidimensional tensors such as linear and convolutional layers, while Adam was used for one-dimensional parameters such as biases and normalisation blocks ^37,38^. Training details are presented in Table 10. No extra weighting of or sampling of positive samples was performed.

**Table 9:** InceptionTime model parameters

Model detail	Value
Inception Blocks	6
Bottleneck Channels	32
Kernel Sizes	9, 19, 39
Channels per Kernel	32

**Table 10:** InceptionTime training details used for training the ensemble members

Training detail	Value
Loss Function	Binary Cross Entropy
Optimiser	Muon and AdamW
Batch Size	64
Initial Learning Rate	0.0037
Learning Rate Scheduler	Cosine Annealing

Implementation was done on a machine with Debian 12.11 in Python 3.12.0 with PyTorch 2.7.1+cu128, and model training and inference were performed on an NVIDIA RTX 5090 with CUDA version 12.9.

### Prospective validation

The prospective validation was conducted on the same platform as the development, using patients enroled in the hospital network between January 1, 2023, and May 1, 2025. Patients from the training dataset were excluded from the validation to prevent information leakage. In contrast to the development set, only one ECG per patient was included in the test set. For patients who had undergone an echocardiographic examination and had an ECG recorded within 14 days, the ECG closest to the echocardiographic examination was included. For the other patients, the earliest recorded ECG was selected. Pairing with NT-proBNP and ICD-10 codes was done similarly to the development phase. From the echocardiographic examinations, EF and input parameters to the H2FPEF risk model ^39^ were extracted. Specifically, the parameters included Body Mass Index (BMI), Doppler echocardiographic estimated pulmonary artery systolic pressure, and Doppler echocardiographic E/e’.

Performance evaluation was conducted under three different labelling strategies, defined in Table 4. These three strategies assess different aspects of HF identification, progressively excluding more patients in exchange for reduced label noise. Initially, for labelling strategy (1), ICD-10 codes were used exclusively, allowing all patients to be included in the test. Subsequently, for labelling strategy (2), NT-proBNP levels were incorporated to validate the diagnosis codes. To minimise data exclusion, we first applied age and sex-adjusted NT-proBNP thresholds defined in Table 5; these are the established thresholds used in clinical practice at the hospital. Then, for labelling strategy (3), the same two-tailed cutoff from development was used, <125 ng/L to be considered healthy and >1000 ng/L for HF, excluding patients in between.

**Fig. 7:**
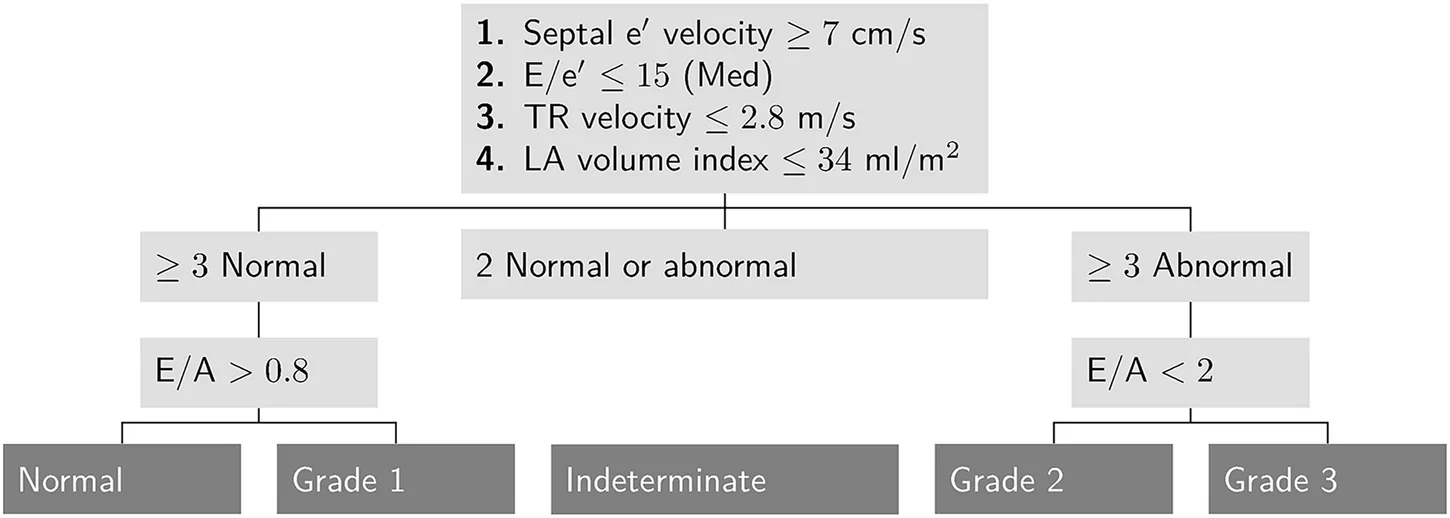
Decision algorithm for diastolic function grading using echocardiographic criteria.

The addition of echocardiography allowed for stratification based on reported examination results, particularly EF. For this subset, evaluations can be conducted on specific phenotypes. Model evaluation using diastolic function grade was inspired by ref. ^15^ that trained AI–ECG to classify diastolic function grade as determined via the algorithm shown in Fig. 7^29^. The algorithm includes five echocardiographic parameters, all of which are not consistently calculated in routine clinical practice. Thus, only patients with all parameters were included in this subgroup analysis.

HFpEF identification was evaluated in detail and was based on several different labelling methods. For all patients with estimated EF > 50%, the H2FPEF score ^39^ was used. The H2FPEF score is a risk assessment tool for HFpEF that profiles independently of NT-proBNP levels. This is particularly valuable as patients with HFpEF, especially those with obesity-related HFpEF ^40^, are known to sometimes exhibit low NT-proBNP levels ^41^. Variables included in the H2FPEF model are BMI, age, filling pressure (Doppler echocardiographic E/e’), and presence of hypertensive heart disease, atrial fibrillation, and pulmonary hypertension^42^. The model used in this study is defined in Table 8, where one notable change is that hypertensive heart disease was identified via ICD-10 codes instead of having 2 or more antihypertensive medicines. The H2FPEF risk model does not include NT-proBNP levels and is widely used in the diagnosis of HFpEF, meaning it is highly relevant to compare our proposed model to the H2FPEF model.

### External validation

External validation was performed using the MIMIC-IV-ECG dataset ^43^, paired with diagnostic codes from MIMIC-IV-ECG-Ext-ICD ^44^ and NT-proBNP values from the MIMIC-IV database ^45^. NT-proBNP was handled consistently with the Ahus cohort using the same labelling strategies, thresholds, and temporal alignment with ECGs. All available ECGs were included without exclusions. The full codebase is publicly available on GitHub, enabling independent reproduction of the dataset processing and model evaluation^46^.

## Supplementary information

Supplementary Information

Supplementary Data 1

Supplementary Data 2

Supplementary Data 3

Supplementary Data 4

Supplementary Data 5

Supplementary Data 6

Supplementary Data 7

## Data Availability

Source data needed to reproduce all figures are provided with this paper (Supplementary Data 1–7). The raw data were collected from Akershus University Hospital, Norway, with ethical approval from Helsedirektoratet. Due to restrictions related to patient privacy, informed consent, and data governance as mandated by Norwegian law, the datasets containing raw data are not publicly available. However, the authors support collaborative scientific analyses and encourage researchers interested in conducting collaborative research or secondary analyses to contact the corresponding authors. Requests for data access will be reviewed individually, and access might require additional ethical approvals in line with relevant guidelines. If the corresponding author does not respond within one week, please get in touch with Ahus Innovation Center for Artificial Intelligence (ai@ahus.no), which manages the hospital's R&D data platform. Entities interested in partnerships or commercial collaborations related to this research should also direct inquiries to ai@ahus.no to establish appropriate data-sharing agreements. The external validation dataset is available through PhysioNet, subject to their data use agreements. The AI model, including training and inference code, as well as the trained weights, is publicly available under the Apache 2.0 license at github.com/Ahus-AIM/Heart-Failure-Detection.
