# Supplementary material for: Heart failure detection in electrocardiograms using artificial intelligence and pragmatic labelling
Source: NPJ Digit Med. 2026 May 19;9:680. doi: 10.1038/s41746-026-02774-4 (PMC13547219; doi:10.1038/s41746-026-02774-4)
Supplement: Supplementary file 1 [file 41746_2026_2774_MOESM1_ESM.pdf]

Supplementary Table 1: Confusion matrix containing all 60 clinically validated cases chosen from the population with EF>50%, NT-proBNP $< 125\,\mathrm{ng/L}$ and no ICD-10 HF diagnosis.

		Validated HFpEF		
		Yes	No	Total
Predicted HFpEF	Yes	24	6	30
	No	3	27	30
	Total	27	33	60

Supplementary Table 2: All 60 clinically validated cases chosen from the population with EF $>$ 50%, NT-proBNP $<$ 125 ng/L, and no ICD-10 HF diagnosis. The list is sorted by model-predicted risk, which can be interpreted as the estimated probability of HF. Some comments were shortened to preserve patient privacy.

**Predicted risk**	**HFpEF**	**Comment**
0.88	Yes	Dilated left atrium
0.88	Yes	Atrial flutter, borderline enlarged left atrium
0.87	Yes	Dilated left atrium and short EDT
0.87	Yes	Dilated left atrium and short EDT
0.84	Yes	High filling pressure
0.8	Yes	Both atria enlarged and myocardial infarction
0.79	No	Slightly elevated EDT and estimated SPAP
0.79	Yes	Acute atrial fibrillation
0.77	Yes	Dilated left atrium and elevated E/e'
0.75	Yes	Atrial fibrillation + pulmonary hypertension
0.73	Yes	Elevated pulmonary pressure and filling pressure in the left ventricle, moderate AI and MI
0.73	Yes	Elevated E/e' and increased pulmonary pressure
0.73	Yes	High filling pressures and severe aortic stenosis
0.72	Yes	Significantly enlarged atria with mild to severe aortic stenosis and pulmonary hypertension
0.68	Yes	Significantly enlarged atria with severe aortic stenosis and hypertrophy
0.65	No	Pacemaker rhythm
0.63	Yes	Significantly enlarged atria on both sides
0.62	Yes	Large left atrium and elevated E/e'
0.6	Yes	Right-sided HF
0.6	No	Mechanical mitral valve with low gradient due to prolapse
0.58	Yes	Mildly enlarged left atrium and elevated pulmonary pressure, right-sided HF
0.58	Yes	Biological aortic valve with high filling pressures
0.58	Yes	Enlarged left atrium with low E/A ratio and dilated ascending aorta
0.56	No	Hypokinesia in the apex after STEMI
0.56	Yes	Diastolic dysfunction with enlarged left atrium
0.55	Yes	LVH with a dilated left atrium
0.55	Yes	Severe aortic stenosis with elevated E/e' and significant atrial enlargement
0.54	No	Acute MI with occluded RCA
0.52	No	Good left-sided function but increased right-sided pressure
0.52	Yes	Dilated left atrium
0.0	No	Athlete's heart with enlarged left atrium
0.0	No	Normal
0.0	No	Normal
0.0	Yes	Systemic sclerosis with probable lung involvement and right-sided HF
0.0	Yes	Enlarged left atrium with low EDT and high E/e'
0.0	No	Normal
0.0	No	Normal
0.0	No	Normal
0.0	No	Three-vessel disease and mild hypokinesia
0.0	No	Normal
0.0	No	Normal but symptomatic coronary artery stenosis that was stented
0.0	No	Normal
0.0	No	Normal
0.0	No	Right-sided HF with elevated pulmonary pressure
0.0	No	Normal
0.0	No	Normal except at aorta ascendens
0.0	No	Normal
0.0	No	Normal
0.0	No	Moderate aortic stenosis
0.0	No	Athlete's heart without signs of HF
0.0	No	Previous SCAD
0.0	No	Small AI
0.0	No	Previous NSTEMI
0.0	No	Non acute SCAD
0.0	No	Mildly enlarged left atrium and borderline elevated pulmonary pressure
0.0	Yes	LVH due to hypertension
0.0	No	Bicuspid valve
0.0	No	Anxiety
0.0	No	Spasm angina
0.0	No	Acute MI

Supplementary Table 3: Comparison between AI-ECG risk and age as a predictor of heart failure. Values are AUROC with 95% confidence intervals.

Evaluated against	AI-ECG risk as predictor	Age as predictor	P-value
Labelling (1)	0.86 (0.847–0.864)	0.67 (0.655–0.676)	< 0.001
Labelling (2)	0.91 (0.900–0.913)	0.71 (0.699–0.718)	< 0.001
Labelling (3)	0.96 (0.951–0.963)	0.83 (0.816–0.838)	< 0.001

Supplementary Table 4: Additional sensitivity analysis, using a version of labelling strategy (3) where $\mathrm{proBNP}_{\mathrm{ever}}$ and $\mathrm{HF}_{\mathrm{ever}}$ are replaced by $\mathrm{proBNP}_{\mathrm{curr}}$ and $\mathrm{HF}_{\mathrm{curr}}$ for the negative (no HF class), resulting in a less strict purification of the negative class. **Using this definition as the ground truth, the model achieved an AUC of 0.925 (95% CI 0.917–0.933) compared to 0.96 (95% CI 0.951–0.963) for labelling strategy (3)**. $\mathrm{HF}_{\mathrm{curr}}$ = HF ICD-10 code within upcoming 60 days of ECG; $\mathrm{HF}_{\mathrm{ever}}$ = any HF ICD-10 code ever; $\mathrm{proBNP}_{\mathrm{curr}}$ = maximum NT-proBNP within $\pm$30 days of ECG, $\mathrm{proBNP}_{\mathrm{ever}}$ = maximum NT-proBNP ever.

	Positive label (HF)	Negative label (no HF)
Labelling	$\mathrm{HF}_{\mathrm{curr}} \wedge (\mathrm{proBNP}_{\mathrm{curr}} > 1000\,\text{ng/L})$	$\neg\,\mathrm{HF}_{\mathrm{curr}} \wedge ((\mathrm{proBNP}_{\mathrm{curr}} < 125\,\text{ng/L}) \vee \text{unknown})$

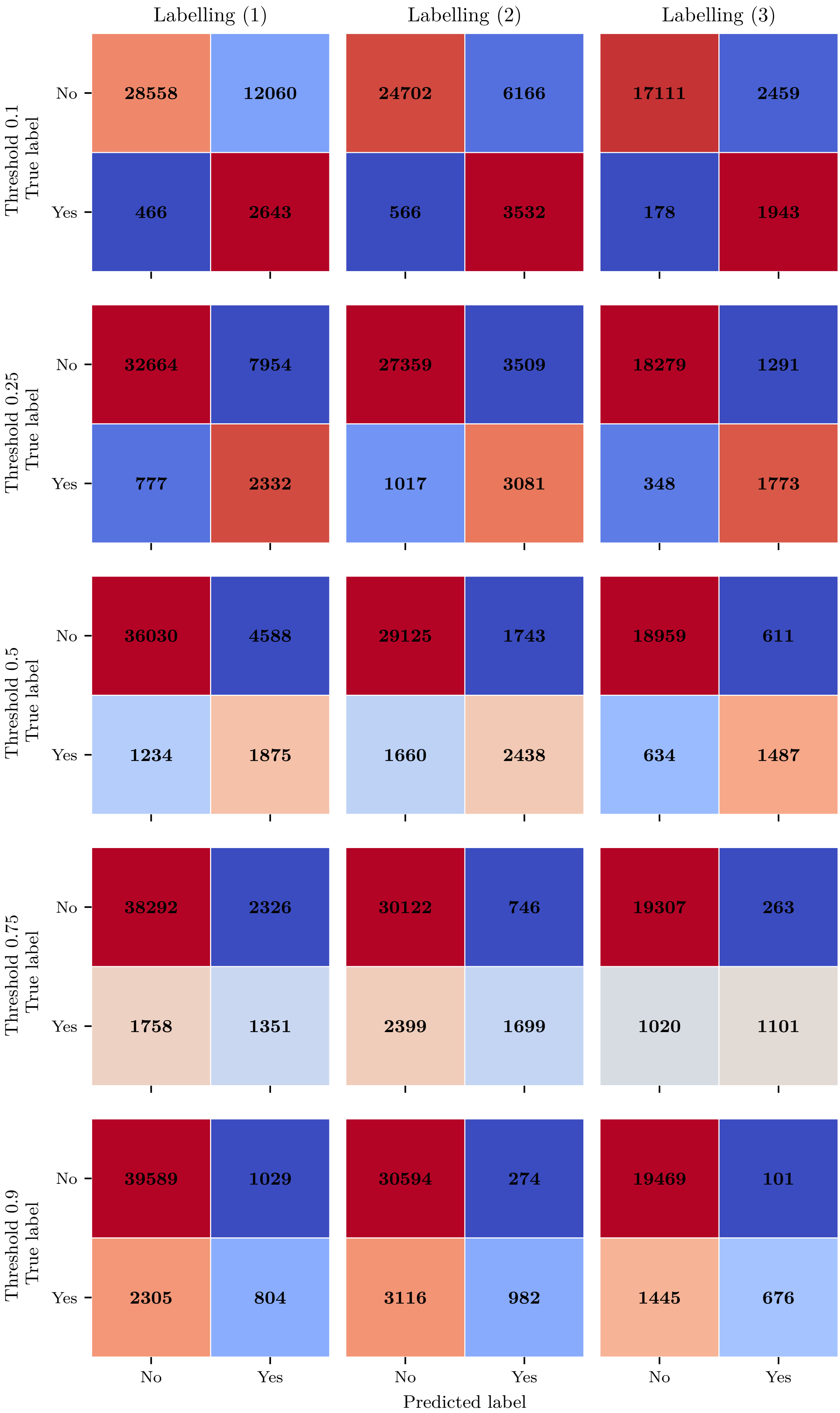

Supplementary Figure 1: Confusion matrices across the Ahus test set for varying thresholds.

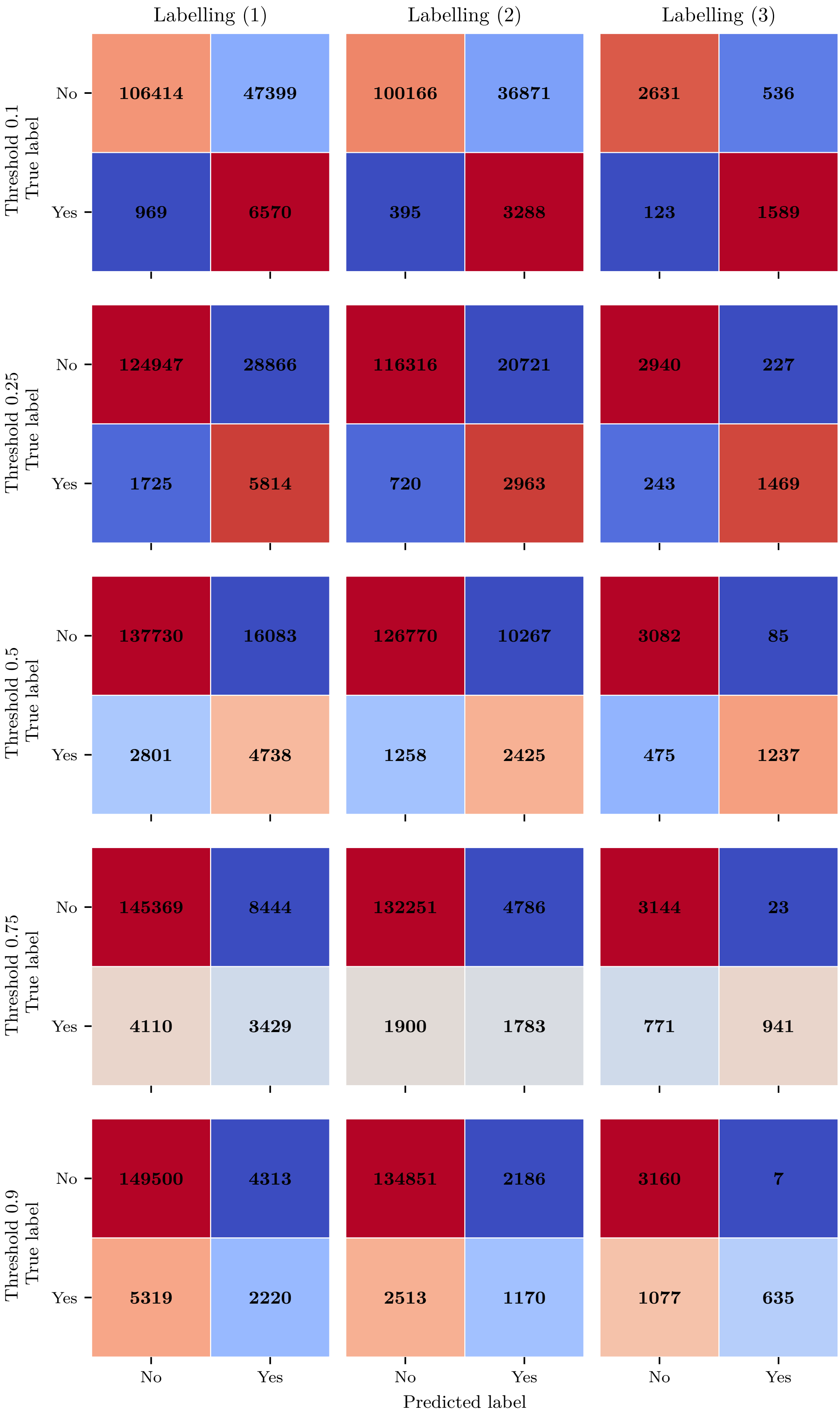

Supplementary Figure 2: Confusion matrices across the MIMIC-IV test set for varying thresholds.

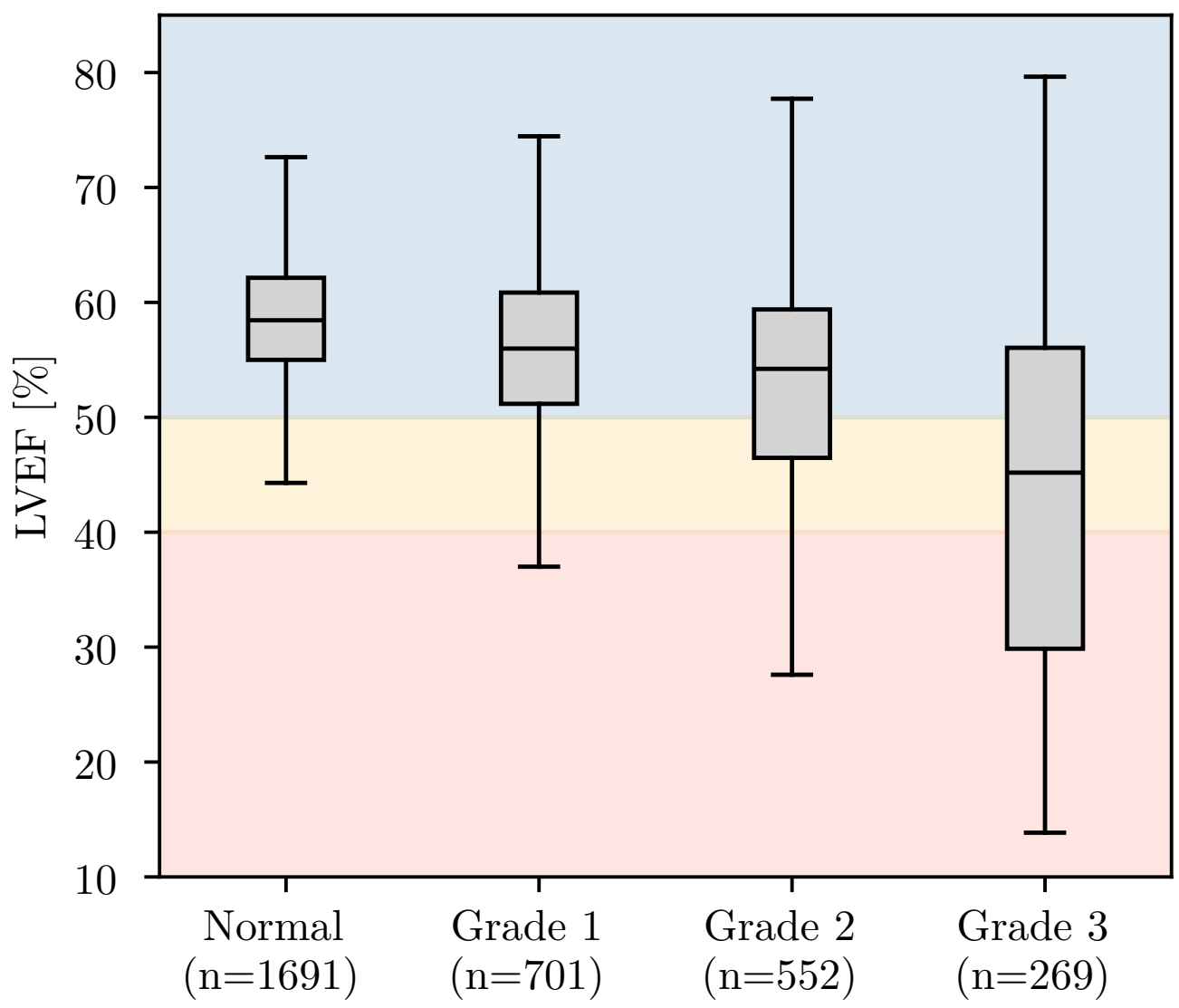

Supplementary Figure 3: Left ventricular ejection fraction stratified by left ventricular diastolic function grade. The background colors correspond to LVEF categories of $< 40\%$, $40 - 50\%$ and $> 50\%$.

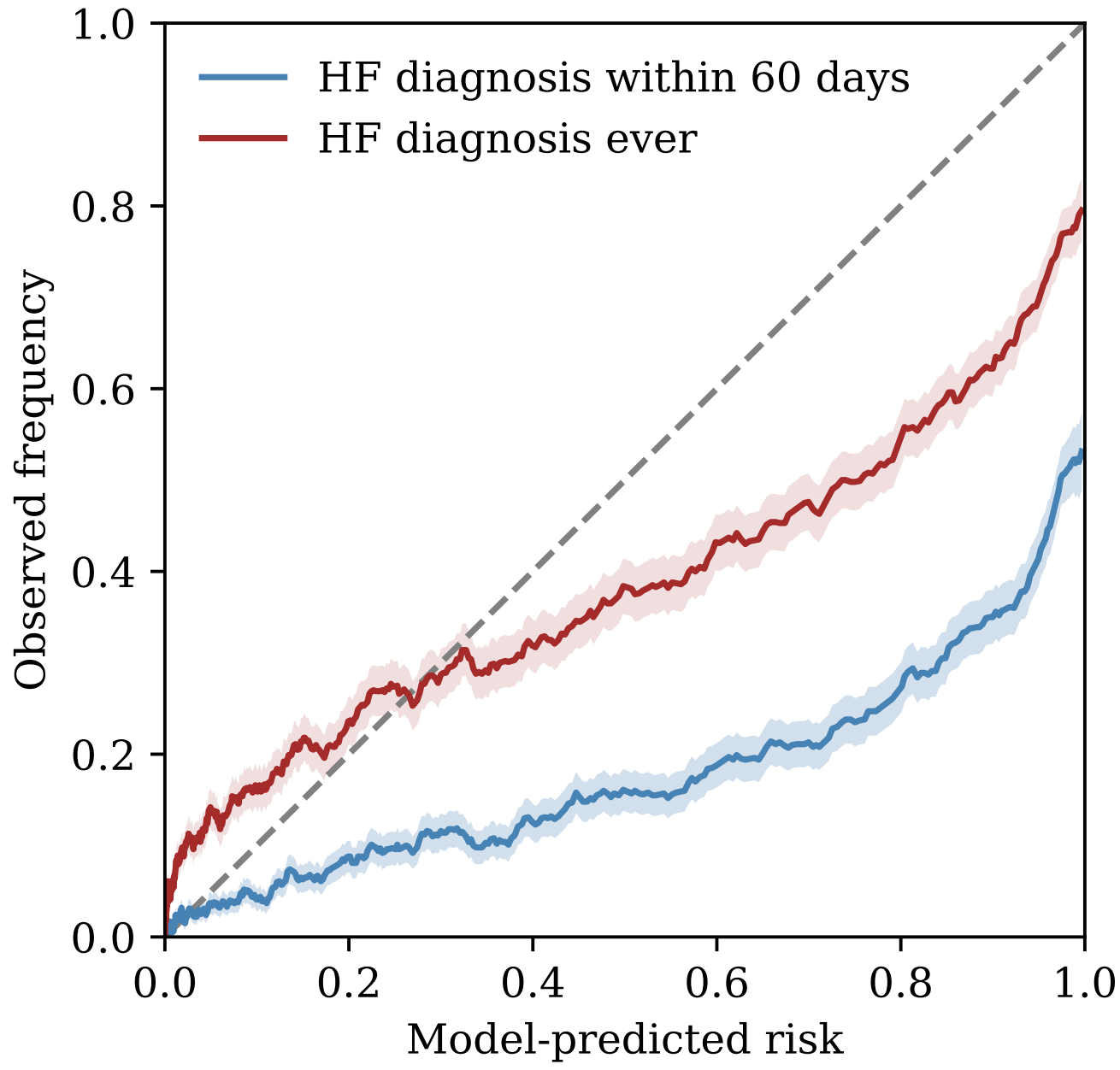

Supplementary Figure 4: Calibration performance of the proposed model when evaluated against ICD-10 coded HF within ± 30 days of the index ECG, and at any time prior to censoring. Observed frequencies are calculated using a moving average window including the 1000 closest neighbours sorted by model-predicted risk, and the shaded area denotes the 95% CI. The Brier score is 0.096 for the shorter time window and 0.12 for the longer time window. Note that ICD-10 coded HF is not equal to the true prevalence of HF due to imperfect coding.
